# A novel MRI-based deep learning–radiomics framework for evaluating cerebrospinal fluid signal in central nervous system infection

**DOI:** 10.3389/fmed.2025.1659653

**Published:** 2025-08-20

**Authors:** Ferhat Cüce, Gökalp Tulum, Muhammed Ikbal Isik, Marziye Jalili, Güven Girgin, Ömer Karadaş, Niray Baş, Berza Özcan, Ümit Savaşci, Sena Şakir, Akçay Övünç Karadaş, Eda Teomete, Onur Osman, Jawad Rasheed

**Affiliations:** ^1^Department of Radiology, Health Science University, Gulhane Training, and Research Hospital, Ankara, Türkiye; ^2^Department of Electrical and Electronics Engineering, Topkapi University, Istanbul, Türkiye; ^3^Department of Artificial Intelligence, Üsküdar University, Istanbul, Türkiye; ^4^Department of Neurology, Muğla Training, and Research Hospital, Muğla, Türkiye; ^5^Department of Neurology, Health Science University, Gulhane Training, and Research Hospital, Ankara, Türkiye; ^6^Department of Infection Disease, Health Science University, Gulhane Training, and Research Hospital, Ankara, Türkiye; ^7^Private Clinic, Ankara, Türkiye; ^8^Department of Classics, University of Michigan, Ann Arbor, MI, United States; ^9^Department of Computer Engineering, Istanbul Sabahattin Zaim University, Istanbul, Türkiye; ^10^Department of Software Engineering, Istanbul Nisantasi University, Istanbul, Türkiye; ^11^Applied Science Research Center, Applied Science Private University, Amman, Jordan; ^12^Research Institute, Istanbul Medipol University, Istanbul, Türkiye

**Keywords:** central nervous system infection, cerebrospinal fluid, brain MRI, Radiomics, deep learning, lumbar puncture, perivascular spaces

## Abstract

**Introduction:**

Accurate and timely diagnosis of central nervous system infections (CNSIs) is critical, yet current gold-standard techniques like lumbar puncture (LP) remain invasive and prone to delay. This study proposes a novel noninvasive framework integrating handcrafted radiomic features and deep learning (DL) to identify cerebrospinal fluid (CSF) alterations on magnetic resonance imaging (MRI) in patients with acute CNSI.

**Methods:**

Fifty-two patients diagnosed with acute CNSI who underwent LP and brain MRI within 48 h of hospital admission were retrospectively analyzed alongside 52 control subjects with normal neurological findings. CSF-related signals were segmented from the ventricular system and sub-lentiform nucleus parenchyma, including perivascular spaces (PVSs), using semi-automated methods on axial T2-weighted images. Two hybrid models (DenseASPP-RadFusion and MobileASPP-RadFusion), fusing radiomics and DL features, were developed and benchmarked against base DL architectures (DenseNet-201 and MobileNet-V3Large) via 5-fold nested cross-validation. Radiomics features were extracted from both original and Laplacian of Gaussian–filtered MRI data.

**Results:**

In the sub-lentiform nucleus parenchyma, the hybrid DenseASPP-RadFusion model achieved superior classification performance (accuracy: 78.57 ± 4.76%, precision: 84.09 ± 3.31%, F1-score: 76.12 ± 6.86%), outperforming its corresponding base models. Performance was notably lower in ventricular system analyses across all models. Radiomics features derived from fine-scale filtered images exhibited the highest discriminatory power. A strict, clinically motivated patient-wise classification strategy confirmed the sub-lentiform nucleus region as the most reliable anatomical target for distinguishing infected from non-infected CSF.

**Discussion:**

This study introduces a robust and interpretable MRI-based deep learning–radiomics pipeline for CNSI classification, with promising diagnostic potential. The proposed framework may offer a noninvasive alternative to LP in selected cases, particularly by leveraging CSF signal alterations in PVS-adjacent parenchymal regions. These findings establish a foundation for future multicenter validation and integration into clinical workflows.

## 1 Introduction

Central nervous system infections (CNSIs) are neurological emergencies that demand prompt and accurate diagnosis to reduce morbidity and mortality. The gold standard for confirming CNSI involves isolating the microbial agent or detecting its antigen in cerebrospinal fluid (CSF), typically via culture or polymerase chain reaction (PCR) analysis following lumbar puncture (LP) (1, 2). However, in clinical practice, the turnaround time for these methods is often inadequate for urgent decision-making. As such, CSF pleocytosis observed on microscopy is frequently used as a proxy to initiate empirical therapy with antibiotics, antivirals, or antifungals (3). Yet, reactive or false-positive pleocytosis may occur—particularly following initial LPs or in immunocompromised patients—raising concerns about overtreatment and diagnostic uncertainty (1).

Furthermore, LP is an invasive procedure with contraindications, including the presence of intracranial mass lesions, bleeding diathesis, spinal malformations, or local infections at the puncture site (2). These factors highlight the need for reliable, noninvasive, and rapid diagnostic tools to support or replace traditional CSF sampling in specific clinical contexts.

MRI plays a vital complementary role in the evaluation of CNSI. Certain imaging patterns—such as asymmetric involvement of the temporal lobe, insula, and cingulum in herpes encephalitis; leptomeningeal enhancement in meningitis; or abscess formation and tuberculous granulomas—may suggest an infectious etiology (4). Nonetheless, normal MRI findings do not exclude infection, and the sensitivity of MRI for viral and bacterial meningitis ranges between 67.4 and 83.3% (5–7). Therefore, neuroimaging alone is insufficient, and there is an urgent demand for advanced image analysis tools that can extract diagnostic information beyond the visual capabilities of radiologists.

Radiomics addresses this gap by converting conventional medical images into high-dimensional quantitative data, capturing subtle image patterns such as intensity, texture, shape, and spatial relationships (8–10). These handcrafted features have shown promise in multiple domains, but their performance can be enhanced when fused with deep learning (DL)–derived features. DL models can automatically learn abstract, hierarchical representations from imaging data, offering complementary insights into disease phenotypes.

Recent studies have demonstrated the efficacy of DL–radiomics fusion models specifically within neurology, such as multimodal neuroimaging feature learning for Alzheimer’s disease diagnosis (11), deep radiomic analysis of MRI data for Alzheimer’s disease classification (12), and fusion of MRI and cognitive assessments for mild cognitive impairment diagnostics (13). Similarly, these approaches have shown promise in distinguishing multiple sclerosis lesions (14) and differentiating Parkinson’s disease patients from healthy individuals using radiomic features from MRI (15) and PET imaging (16). Additionally, deep learning radiomic frameworks have been effectively used for predicting hemorrhage progression in intracerebral hemorrhage (17), forecasting outcomes after acute ischemic stroke (18), and diagnosing temporal lobe epilepsy through FDG-PET imaging (19).

Despite the growing interest in end-to-end deep learning pipelines, current evidence suggests that combining DL with handcrafted radiomics yields more interpretable and robust results especially in datasets with limited sample sizes (20–22). Consequently, standardization initiatives now recommend best practices for preprocessing, feature selection, and model validation to improve reproducibility across institutions (23).

In this study, we propose a hybrid DL–radiomics framework for classifying infected versus non-infected CSF regions in patients with suspected CNSI. We focus on two anatomical targets: the ventricular system and the sub-lentiform nucleus parenchyma, including the perivascular spaces (PVSs), which are implicated in glymphatic CSF circulation. We hypothesize that the fusion of radiomic descriptors and DL-based spatial features can enable noninvasive discrimination of CSF infection patterns, thereby supporting earlier diagnosis and potentially reducing the reliance on lumbar puncture.

## 2 Methods and materials

### 2.1 Patient

The local ethics committee approved this retrospective study, and written consent was waived.

This retrospective study included patients diagnosed with CNSI who underwent brain MRI as part of their routine clinical work-up between 2017 and 2024. Fifty-two patients in the infection group were diagnosed with acute bacterial, viral and aseptic meningitis based on a combination of clinical presentation (e.g., fever, headache, neck stiffness), CSF analysis, and microbiological testing. Importantly, none of the included patients met the diagnostic criteria for encephalitis or meningoencephalitis, and there were no findings suggestive of parenchymal involvement (such as diffusion restriction, edema, or signal abnormalities and contrast enhancement in the brain parenchyma) on MRI. Mild to moderate leptomeningeal enhancement was observed in the majority of cases on post-contrast T1-weighted images, which was consistent with active meningeal inflammation. No significant ventriculitis, abscess formation, or hydrocephalus was detected. Clinically, patients presented primarily with headache and fever, and none exhibited focal neurological deficits, altered mental status, or seizures at the time of imaging. This strict inclusion criterion ensured a clinically and radiologically homogeneous infection cohort, thereby allowing a focused evaluation of CSF-related signal features in isolated meningitis and minimizing potential confounding from parenchymal disease.

All patients diagnosed with CNSI underwent an LP on the day of admission and had brain MRIs performed within the first 48 h after being admitted to the hospital. We excluded patients who did not undergo LP, had no brain MRI, had MRIs taken more than 48 h after treatment commenced.

The control group consisted of 52 patients with chronic headaches with normal neurological examinations and normal brain MRI reports. A total of 104 patients, including both the patient and control groups, were included in the analysis.

### 2.2 Imaging parameters

All brain MRIs were performed on a Philips 3 T imaging system with a dedicated head coil. All studies included axial plane fat-saturated fast spin eco T2-weighted sequence with time repetition (TR): 2,600–5,600 millisecond (ms), time echo (TE): 70–90 ms, echo train length (ETL):10–12. The slice thickness was 5 millimeters (mm). To accurately evaluate subtle cerebrospinal fluid (CSF)-specific signal alterations and to minimize inadvertent segmentation errors arising from CSF flow artifacts, pre-contrast T2-weighted images were exclusively utilized in this study. T2-weighted imaging was selected for its inherent sensitivity and superior contrast resolution regarding fluid characteristics, enabling precise and artifact-aware segmentation of CSF regions. On the other hand, sequences such as T1-weighted, post-contrast T1-weighted, FLAIR, and diffusion-weighted images (DWI) were deliberately excluded. T1-weighted and post-contrast sequences primarily emphasize anatomical structures and contrast-enhanced parenchymal or meningeal lesions, providing limited utility in isolated CSF analysis without parenchymal involvement. Likewise, FLAIR imaging suppresses CSF signals, inherently limiting its applicability for dedicated CSF signal assessment. DWI is particularly sensitive to acute parenchymal lesions, but since our study specifically excluded patients with parenchymal abnormalities, its inclusion was not considered beneficial. Since no 3D modeling was employed in our study, the slice thickness of 5 mm did not constitute a significant limitation for our analysis. This selective approach ensured methodological consistency and enhanced reliability in analyzing isolated CSF-related radiomic and deep learning features.

### 2.3 Semi-automated segmentation procedure

**Figure 1:**
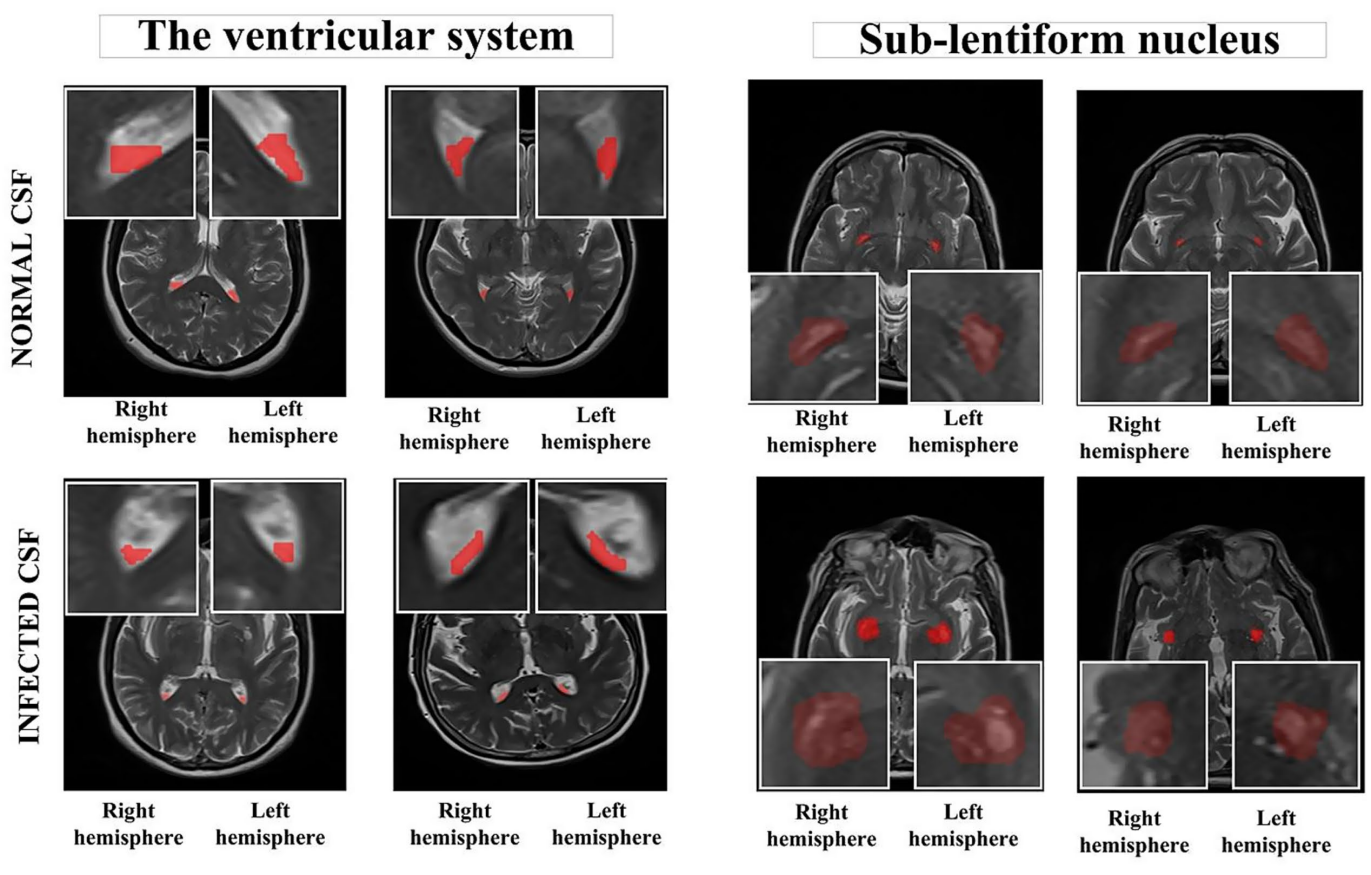
Segmented anatomical regions from the ventricular system and the sub-lentiform nucleus in both right and left hemispheres. The top row illustrates two representative cases from the control group with normal CSF, while the bottom row presents two cases from the CNSI group with infected CSF. Red-highlighted regions indicate the manually segmented areas used for radiomics feature extraction. The bounding boxes were generated as standardized input patches for deep learning models. All images are derived from T2-weighted MRI sequences. CNSI, Central Nervous System Infection; CSF, Cerebrospinal fluid.

Upon consensus, two independent radiologists determined the slices in the axial planes of T2-weighted images. Subsequently, MRI images were stored in the DICOM file format and imported to the ManSeg (v.2.7d) software (24). Initially, the radiologists focused on segmenting the CSF signal in both the upper and posterior sections of the lateral ventricles’ lumen, avoiding areas with visible flow artifacts. Next, to reduce the risk of missing any subtle, instantaneous changes in the normal CSF flow signal, they separately segmented the parenchyma of the sub-lentiform nucleus, which includes the perivascular spaces (PVSs) supplied by the lenticulostriate arteries. Sub-lentiform nucleus parenchyma with the PVSs would effectively represent the features of the CSF, including its contents. For each patient, the lateral ventricles’ lumen and the parenchyma of the sub-lentiform nucleus were segmented bilaterally. For the segmentation of suspicious regions, the radiologists roughly delineated the boundaries of the regions of interest independently, and the segmentation process was then performed automatically using the active contour algorithm (25). Final consensus segmentation masks were obtained after resolving discrepancies through joint review. Inter-observer agreement was assessed retrospectively on a randomly selected subset of 10 patients. Mean Dice similarity coefficients were 0.92 ± 0.03 for ventricular regions and 0.91 ± 0.04 for sub-lentiform parenchyma. Figure 1 depicts samples of infected CSF and normal CSF on T2-weighted images, respectively.

### 2.4 Feature extraction

Radiomics features were extracted from the segmented regions on both the native T2-weighted MRI images and three Laplacian-of-Gaussian (LoG)–filtered counterparts generated with kernel sizes of 3 × 3 × 1 (fine), 5 × 5 × 2 (medium), and 7 × 7 × 3 (coarse). While 2D morphological features were derived solely from the original T2 images, both first-order and second-order statistical features, including those from gray level co-occurrence matrix (GLCM), gray level size zone matrix (GLSZM), gray level run length matrix (GLRLM), neighboring gray-tone difference matrix (NGTDM), and gray level dependence matrix (GLDM) were extracted from all image sources. A comprehensive list of the extracted features is presented in Table 1, comprising a total of 378 features.

**Table 1:** The description and the total number of radiomics features.

Image Type	Feature Class	Number of features	Total number of features
Original image	1. First order statistics	17	102
	2. 2D shape features	9	
	3. Gray level co-occurrence matrix (GLCM) features	24	
	4. Gray level size zone matrix (GLSZM) features	16	
	5. Gray level run length matrix (GLRLM) features	16	
	6. Neighboring gray tone difference matrix (NGTDM) features	5	
	7. Gray level dependence matrix (GLDM) features	14	
Log filter (FINE, MEDIUM, COARSE PATTERNS)	1. First order statistics	51	276
	2. Gray level co-occurrence matrix (GLCM) features	72	
	3. Gray level size zone matrix (GLSZM) features	48	
	4. Gray level run length matrix (GLRLM) features	48	
	5. Neighboring gray tone difference matrix (NGTDM) features	15	
	6. Gray level dependence matrix (GLDM) features	42	

### 2.5 Classification methodology

First, the region of interest (ROI) images and their corresponding radiomics features were imported. For the deep-learning analysis, each segmented region was centrally cropped into a 32 × 32 pixel patch, which was then resized to 224 × 224 pixels using bicubic interpolation. A patient-based 5-fold cross-validation (CV) approach was employed, ensuring that each patient’s ROI images and associated radiomics data remained grouped during the splitting process. One-fold was allocated as the test set, while the remaining folds were used for training and validation. Feature selection was conducted solely on the radiomics features derived from the training and validation sets. From a total of 378 radiomics features, the top 50 most discriminative features were selected using a filter-based approach. Subsequently, the training and validation sets were split into an internal 3-fold cross-validation (CV) to divide them into training and validation subsets further. Data augmentation techniques, including rotation, zooming, translation, and flipping, were applied to enhance the diversity of the training data.

In our preliminary analyses, we evaluated several advanced architectures, including Swin Transformer, Vision Transformer (ViT), and attention-based networks. However, these approaches yielded poor performance and instability due to the relatively limited size of our dataset. Therefore, DenseNet-201 and MobileNet-V3Large were selected as robust baseline architectures, given their known ability to generalize well on smaller datasets and their compatibility with our hybrid feature fusion strategy.

**Figure 2:**
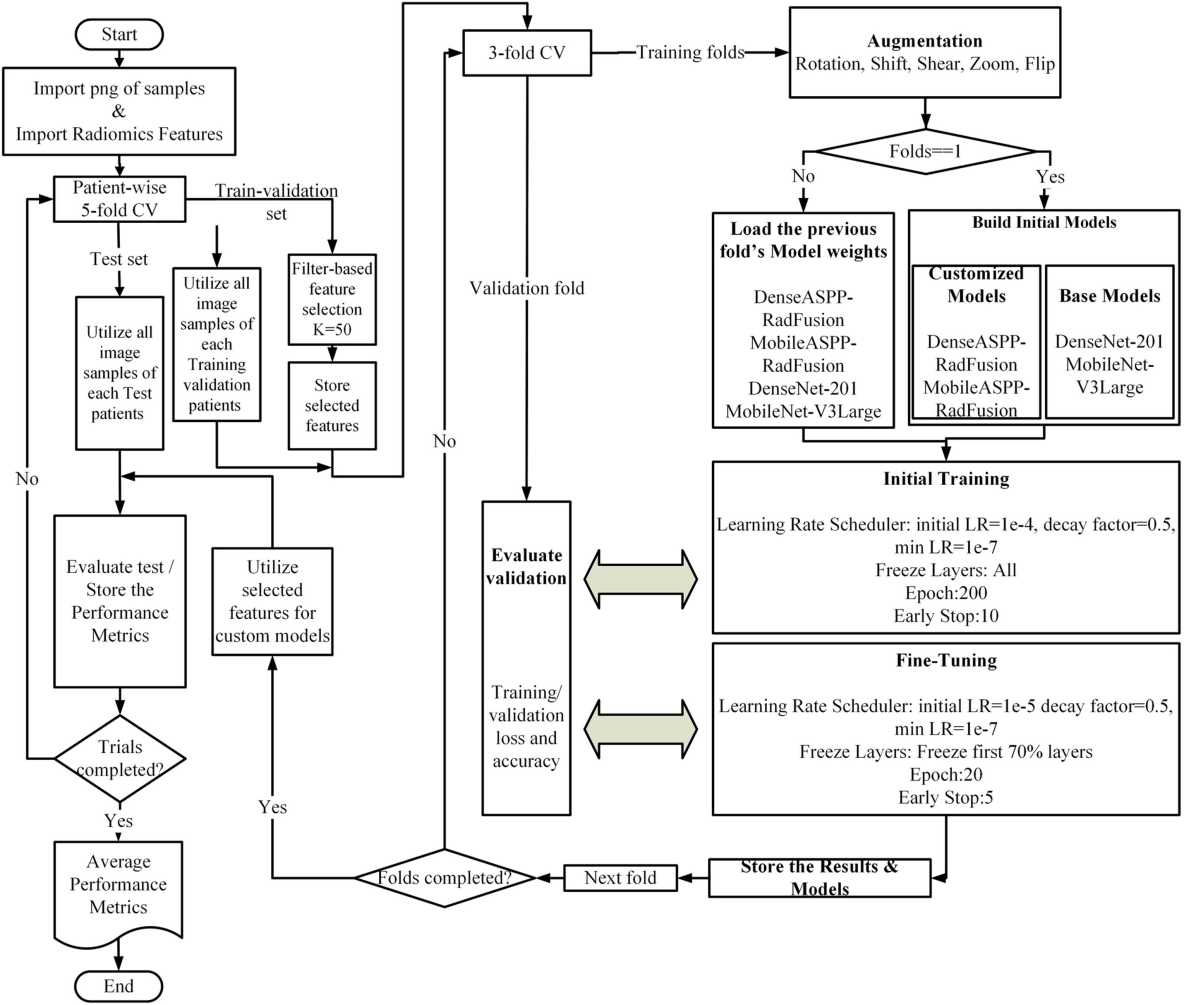
Overview of the proposed classification framework.

Model training was conducted in two phases. For the first outer fold, both the customized models DenseASPP-RadFusion and MobileASPP-RadFusion and the base models DenseNet-201 (26) and MobileNet-V3Large (27) were initialized from scratch. For the remaining folds, the weights from the previous fold were loaded to continue training. During the initial training phase, the learning rate was set to 1e-4 with a reduction factor of 0.5 and a minimum learning rate of 1e-7. Training proceeded for up to 200 epochs, with early stopping implemented after 10 epochs. During the fine-tuning phase, the learning rate was reduced to 1e-5, and the first 70% of the layers were frozen. Training was conducted for 20 epochs, with early stopping triggered after five epochs. These hyperparameters were empirically determined based on iterative experimentation within the internal training-validation splits to minimize overfitting. No hyperparameter tuning was performed on the external test sets. Throughout the process, training and validation loss, as well as accuracy metrics, were monitored. At the end of each fold, model weights and performance metrics were saved. During the testing phase, the feature selection obtained from the outer fold was applied to the test set, and model performance was evaluated using standard metrics, including accuracy, precision, recall, and F1-score. Finally, the results from all five folds were reported as mean ± standard deviation for each performance metric. Figure 2 depicts the flowchart of the classification process.

In the baseline architecture, models such as DenseNet-201 and MobileNet-V3Large were employed as feature extractors. These base models processed the input MRI images to generate feature maps, which were subsequently passed through a global average pooling layer, followed by a fully connected layer with 256 neurons and a dropout layer (rate = 0.3), leading directly to the classification output. In contrast, the proposed fusion models were designed to integrate both deep image features and handcrafted radiomics features. In the image branch of the proposed models, the backbone feature map was processed through five parallel paths. Four of these paths constituted the Atrous Spatial Pyramid Pooling (ASPP) module, employing 3 × 3 convolutions with dilation rates of 1, 6, 12, and 18, each followed by batch normalization and ReLU activation, producing four parallel 7 × 7 × 512 feature maps. The fifth path was designed to inject global contextual information by applying global average pooling to the backbone feature map (resulting in 1 × 1 × 1920), followed by a 1 × 1 convolution with 512 filters, and then bilinear upsampling to reach a size of 7 × 7 × 512. All five outputs were concatenated to form a unified representation of size 7 × 7 × 2,560 and then compressed via a 1 × 1 convolution with 512 filters.

**Figure 3:**
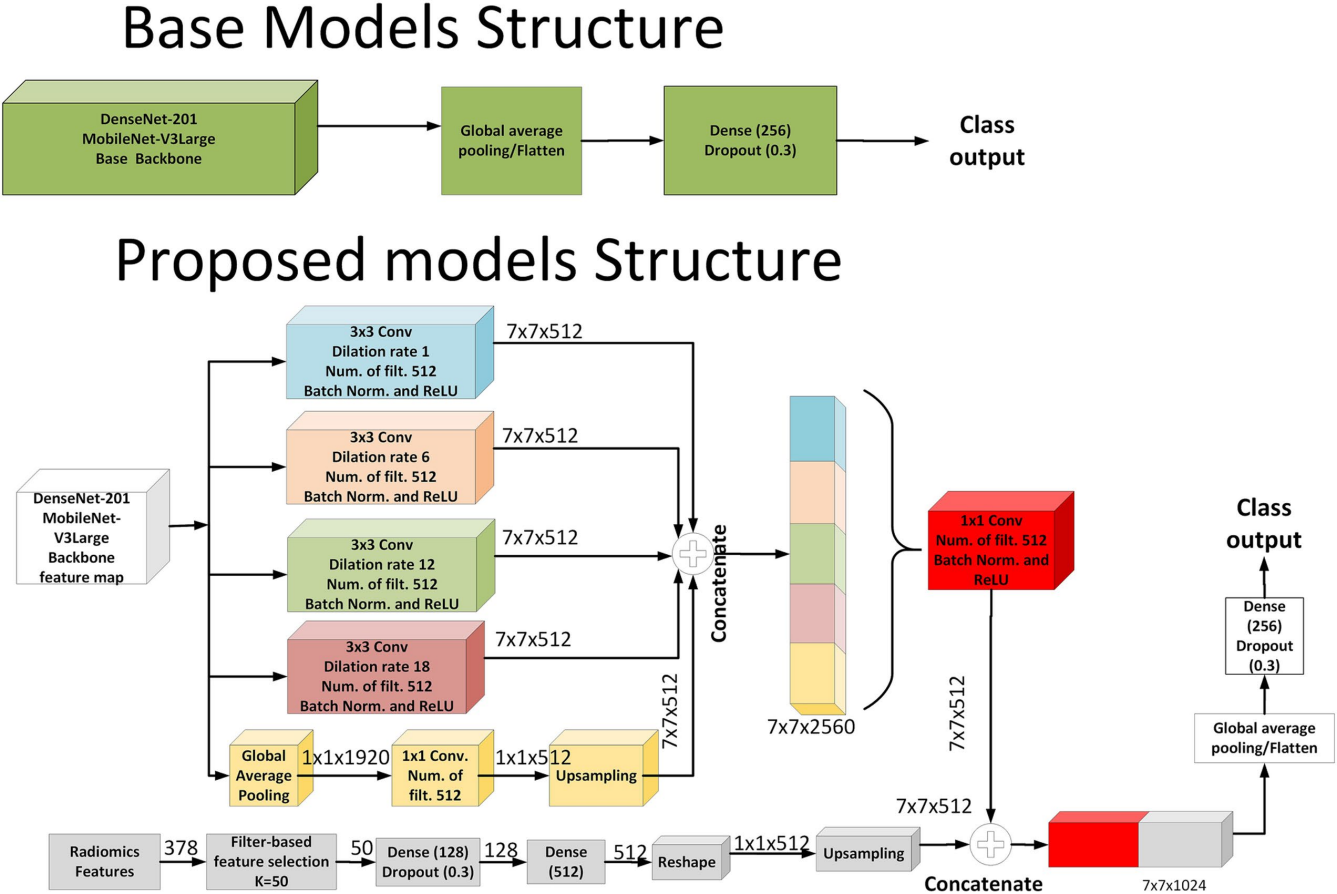
Schematic representation of the baseline and proposed model architectures.

In parallel to the image pathway, radiomics features were processed through a separate branch. A total of 378 radiomics features were extracted and reduced to 50 using filter-based feature selection. These selected features passed through two fully connected layers [Dense (128) and Dense (512)] with dropout, reshaped into a 1 × 1 × 512 tensor and then upsampled to 7 × 7 × 512 to match the spatial resolution of the image features. Finally, the outputs from both the image and radiomics branches were concatenated along the channel axis, forming a 7 × 7 × 1,024 fused representation. This combined feature map was subjected to global average pooling, followed by a Dense (256) layer with dropout, and terminated with a softmax classification layer. This architecture effectively captured both spatial and contextual information from MRI data, enriched by complementary radiomics descriptors. As illustrated in Figure 3, the proposed model architecture integrates both ASPP-enhanced image features and spatially fused radiomics features. The implementation code for the proposed MRI-based deep learning–radiomics framework is publicly available at: https://github.com/DrGokalpTulum/MRI-Based-Deep-Learning-Radiomics-Framework-for-Evaluating-Cerebrospinal-Fluid-Signal-.git.

## 3 Results

In the CNSI group, 55.7% (*n* = 29) of the patients were male, 44.3% (*n* = 23) were female, and the mean age was 43.5 ± 22.5 years. In the control group, 33.9% (*n* = 18) of the patients were male, 66.1% (*n* = 34) were female, and the mean age was 46.7 ± 11 years.

The CSF analysis was performed on the patient’s admission to the health institution. The macroscopic appearance of the CSF, the amount of CSF glucose and protein, pleocytosis in microscopy, and the presence of microorganisms in the Gram stain were evaluated. High CSF protein, low glucose, leukocyte count of 100 or more cells/mm3, and neutrophil predominance are evaluated as bacterial meningitis; normal CSF glucose, borderline high protein levels, and lymphocytes being the predominant cell in the cell count were evaluated as viral meningitis; normal CSF findings were accepted as aseptic meningitis.

According to early biochemical and microscopy results, bacterial meningitis was observed in 37 patients, viral meningitis in 14 patients, and CSF findings of 1 patient were evaluated as aseptic meningitis. While no culture medium growth was detected in the CSF of 24 patients, Streptococcus was detected in 5 patients, *E. coli* in 3 patients, Brucella in 2 patients, Acinetobacter in 1 patient, Neisseria in 1 patient, and Proteus in 1 patient, according to CSF culture results. Varicella Zoster Virus PCR positivity was detected in the CSF of two patients. Based on clinical and laboratory results in the patient group, antimicrobial treatment for CNSI was empirically started. After the diagnosis of the agent was confirmed by culture, PCR, and serology, treatment revision was performed with de-escalation in three patients.

During the 5-fold outer cross-validation, a total of 378 radiomics features were subjected to feature selection, and the top 50 features were retained in each fold. Across all folds, a total of 92 unique features were selected. Among these, 20 features were consistently selected in all five folds, indicating strong discriminative capacity. These high-frequency features primarily originated from the Laplacian of Gaussian (LoG) filtered MRI with fine kernels (2 mm). In particular, features such as Energy, Maximum, Range, Long Run Emphasis, and High Gray Level Zone Emphasis repeatedly appeared across all folds.

Additionally, 16 features appeared in four folds and five features in three folds, most of which stemmed from LoG-filtered MRI with medium kernels (4 mm) or original T2-weighted images. These consistently selected features highlight the critical role of multiscale texture descriptors in capturing the heterogeneity of cerebrospinal fluid regions. Detailed feature selection results, including Feature Name, Image Source, Feature Class, and Frequency, are provided in the Supplementary file.

Upon investigating the classification results, the proposed fusion models (DenseASPP-RadFusion and MobileASPP-RadFusion) demonstrate improvements over their corresponding base architectures (DenseNet-201 and MobileNet-V3Large) in the sub-lentiform nucleus parenchyma region. DenseASPP-RadFusion achieved the highest mean accuracy (78.57 ± 4.76%) and precision (84.09 ± 3.31%), with relatively low standard deviations, indicating both high performance and consistency across folds. Although MobileASPP-RadFusion yielded the highest mean recall (77.05 ± 14.82%), the associated standard deviation was relatively large, suggesting instability in sensitivity across different validation folds.

In contrast, none of the models showed strong classification capability in the ventricular system. Accuracy values remained between 57.26 and 60.52%, while F1-scores were notably lower, particularly for DenseASPP-RadFusion (53.46 ± 11.04%) and DenseNet-201 (49.37 ± 15.32%). Moreover, the standard deviations in recall for all models were high (ranging from 12.94 to 20.66%), indicating a lack of reliability in detecting true positives in ventricular-level CSF signals.

**Figure 4:**
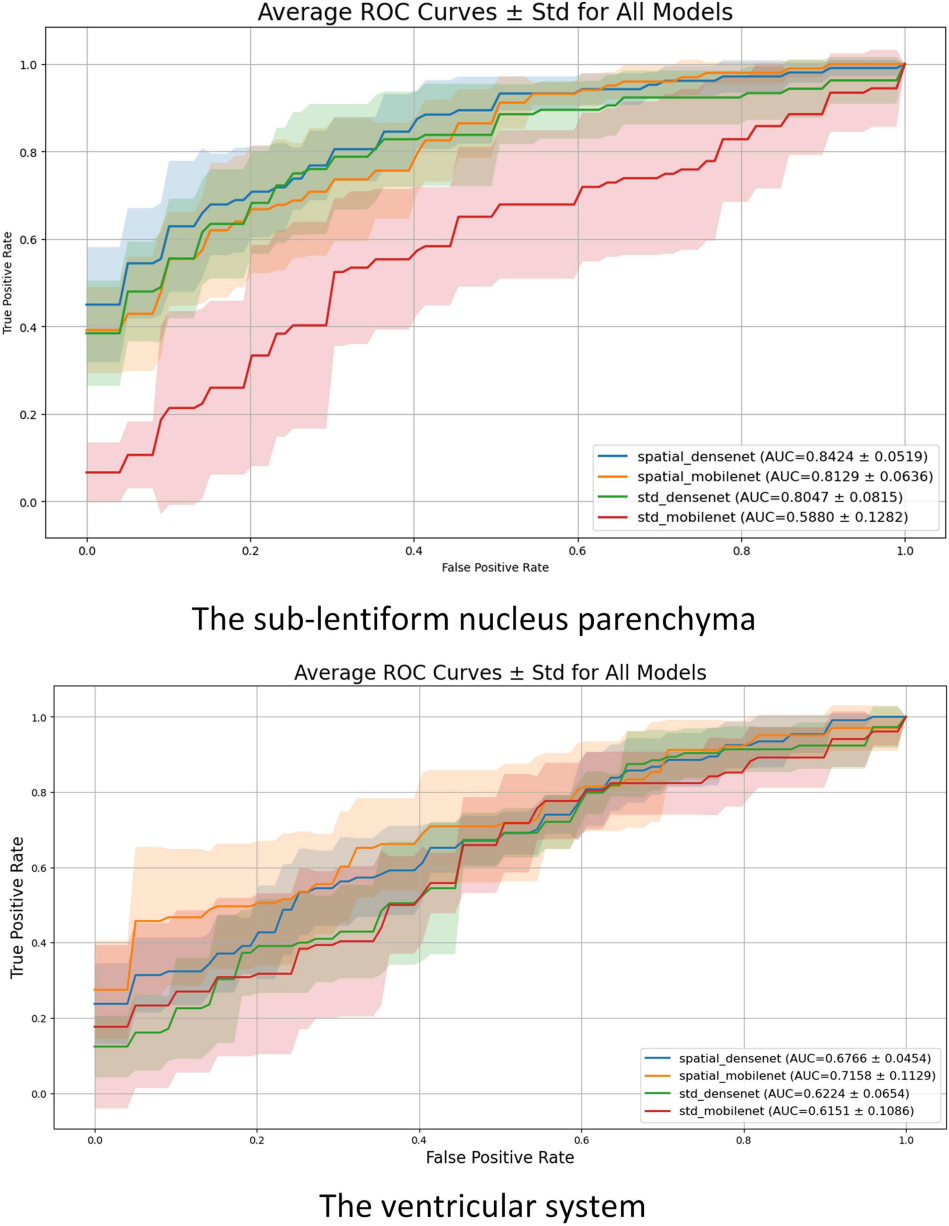
Mean ROC curves with standard deviation (shaded regions) for each model across the 5-fold cross-validation. The upper plot illustrates results for the sub-lentiform nucleus parenchyma with PVSs. The lower plot presents results for the ventricular system. Legend entries include average AUC ± standard deviation for each model.

The results show that the sub-lentiform nucleus parenchyma with PVSs provides more stable and discriminative information for classification tasks compared to the ventricular system. The performance of the models was statistically significantly different (*p* < 0.05). Detailed performance metrics for all models and anatomical regions are presented in Table 2, while the corresponding ROC curves are illustrated in Figure 4.

**Table 2:** Performance metrics (mean ± std) of all models.

Evaluation Area	Model	Accuracy (%)	Precision (%)	Recall (%)	F1 Score (%)
Performance metrics for the sub-lentiform nucleus parenchyma	DenseASPP-RadFusion	78.57 ± 4.76	84.09 ± 3.31	70.00 ± 10.93	76.12 ± 6.86
	MobileASPP-RadFusion	74.40 ± 2.28	73.66 ± 4.41	77.05 ± 14.82	74.42 ± 6.66
	DenseNet-201	73.81 ± 8.01	79.72 ± 5.18	62.96 ± 14.04	70.03 ± 10.52
	MobileNet-V3Large	52.98 ± 9.20	55.66 ± 13.09	76.14 ± 27.62	60.84 ± 6.49
Performance metrics for the ventricular system	DenseASPP-RadFusion	60.52 ± 4.87	64.65 ± 8.03	47.64 ± 17.02	53.46 ± 11.04
	MobileASPP-RadFusion	59.07 ± 8.63	58.14 ± 8.86	70.18 ± 26.40	61.58 ± 13.00
	DenseNet-201	57.26 ± 7.05	58.54 ± 13.48	44.64 ± 20.66	49.37 ± 15.32
	MobileNet-V3Large	59.62 ± 5.06	56.69 ± 4.68	77.36 ± 12.94	65.28 ± 7.26

The upper section presents results for the sub-lentiform nucleus parenchyma with PVSs, while the lower section shows results for the ventricular system. Metrics evaluated across 5-fold cross-validation for each model.

To complement the fold-level evaluation, patient-wise classification performance was also assessed under clinically motivated assumptions. To assess patient-level diagnostic performance under clinical assumptions, strict patient-wise accuracy was calculated separately for each class (infection and control) across all outer folds. Since each patient had two separate ROIs from the right and left sub-lentiform nucleus levels, the following decision rules were applied: for infection cases (class 1), a prediction was considered correct if at least one of the two ROIs was classified as infected, reflecting a clinically cautious approach to minimize false negatives. Conversely, for control cases (class 0), a prediction was deemed correct only if both ROIs were classified as non-infected, ensuring stricter criteria for healthy labeling. This binary patient-wise accuracy was computed per case and averaged within each fold for all models.

**Figure 5:**
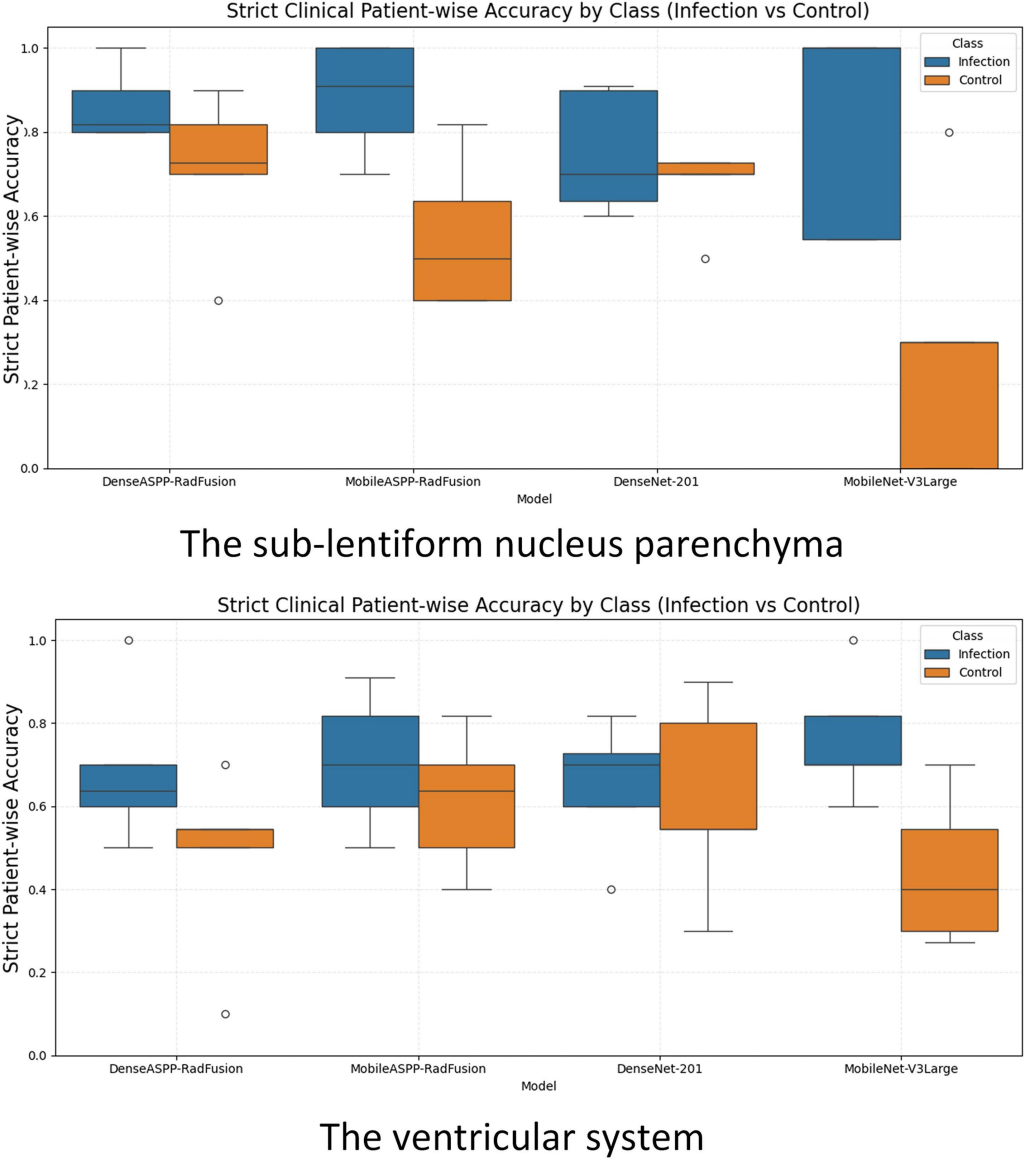
Box plots illustrating strict clinical patient-wise accuracy for CNSI and control classes across all models, evaluated separately for the sub-lentiform nucleus parenchyma (top) and the ventricular system (bottom). CNS, Central nervous system infection.

Figure 5 presents box plots illustrating the distribution of strict patient-wise accuracy values for each model, separately for the sub-lentiform nucleus parenchyma and ventricular system. In the sub-lentiform nucleus parenchyma with PVSs, the proposed model DenseASPP-RadFusion yielded the most stable and accurate performance, with infection class accuracies tightly clustered within the 80 to 90% interquartile range and control accuracies between 70 and 80%, both showing low interfold variability. Similarly, DenseNet-201 achieved high median values, though with a slightly wider spread in the control group. Notably, both models exhibited limited presence of outliers, suggesting consistency in predictions across patient subsets.

On the other hand, MobileNet-V3Large exhibited high variability and lower median accuracy, particularly for control patients. Its control group performance distribution dropped to a lower interquartile range (below 60%) and revealed several outliers, reflecting instability across folds. MobileASPP-RadFusion demonstrated acceptable median accuracy but higher dispersion, particularly in control cases, indicating less consistent generalization across folds.

In the ventricular system, all models demonstrated lower and more dispersed accuracy distributions, indicating reduced reliability in this anatomical region. For instance, although MobileNet-V3Large achieved reasonable infection accuracy, its control classification remained weak and inconsistent. MobileASPP-RadFusion and DenseNet-201 exhibited moderate accuracy with noticeably higher standard deviations, particularly in control predictions, highlighting the challenge of robust CSF signal interpretation in ventricular regions. The broader interquartile ranges and frequent outliers in the ventricular plots underscore the inconsistency of model behavior in this region. These findings further reinforce that the sub-lentiform nucleus parenchyma with PVSs provides a more clinically reliable classification, both at the level of the fold and the patient.

Under this realistic criterion, the proposed fusion models demonstrated high stability and accuracy, particularly in the sub-lentiform nucleus. By contrast, all models showed reduced and inconsistent performance in ventricular CSF classification, further underscoring the diagnostic limitations of relying solely on ventricular analysis. Discordant predictions between left and right sub-lentiform nucleus evaluations occurred in 22.6 ± 3.1% for DenseASPP-RadFusion, 30.4 ± 6.0% for MobileASPP-RadFusion, 29.9 ± 4.7% for DenseNet-201, and 39.5 ± 5.9% for MobileNet-V3Large, indicating varying levels of stability in bilateral predictions.

## 4 Discussion

In this study, we developed and evaluated a novel MRI-based deep learning–radiomics framework to classify CSF signals in patients with acute CNSIs. Our findings demonstrate that the fusion of handcrafted radiomic descriptors with DL features enables more accurate and reliable classification of infected versus non-infected CSF, particularly when analyzing the sub-lentiform nucleus parenchyma region. These results offer promising evidence for the utility of noninvasive imaging-based diagnostics as a potential complement or alternative to LP in selected clinical contexts.

Despite their central role in CNSI diagnosis, CSF analyses via LP remain invasive and carry procedural risks, including herniation, hemorrhage, or infection—especially in patients with intracranial mass lesions or bleeding disorders (1–3). Moreover, pleocytosis, often used as a surrogate marker of infection, may occasionally yield false-positive results, especially after repeated LPs or in immunocompromised individuals (1). These limitations necessitate the development of alternative diagnostic strategies that are rapid, noninvasive, and reproducible.

While MRI has proven valuable in detecting certain CNSI patterns—such as temporal lobe involvement in herpes encephalitis or leptomeningeal enhancement in meningitis—it lacks sufficient sensitivity to reliably detect all cases, particularly in early or ambiguous presentations (4–7). In our study, conventional visual inspection of ventricular CSF signals on MRI did not provide sufficient discriminatory power to distinguish infected from non-infected fluid. This is likely due to the inherent signal homogeneity and dynamic flow of CSF in the ventricles, which limits the effectiveness of static image-based analysis.

Indeed, previous AI-based studies evaluating body fluid segmentation—such as pleural or synovial effusions—have reported promising results (28, 29). However, these studies primarily focused on relatively static fluids that exhibit well-defined boundaries and textural consistency. CSF, on the other hand, is in constant motion, and its flow-dependent signal properties pose substantial challenges for conventional image segmentation and classification.

To address these limitations, our study focused on the sub-lentiform nucleus parenchyma, specifically targeting regions that include perivascular spaces (PVSs)—components of the glymphatic system that mediate convective CSF flow from penetrating arteries into the interstitial space. Unlike the ventricular system, these parenchymal regions are less affected by flow artifacts and may reflect more stable and informative imaging features. Additionally, inflammation in adjacent brain parenchyma during CNSI—though often invisible on routine MRI—may alter tissue texture and contribute to detectable radiomic changes.

Our results strongly support this hypothesis. The hybrid DenseASPP-RadFusion model, which integrates multiscale radiomics with spatially resolved DL features, achieved a mean classification accuracy of 78.6% in the sub-lentiform nucleus region—substantially outperforming both its base architecture (DenseNet-201) and all models applied to the ventricular system. Features derived from Laplacian of Gaussian (LoG)–filtered images, particularly with fine kernels (2 mm), contributed most significantly to model performance, suggesting that subtle intensity variations in the CSF-parenchyma interface are key discriminative elements.

Furthermore, we applied a clinically grounded, strict patient-wise classification strategy, wherein a diagnosis of infection was accepted if either hemisphere exhibited an infected CSF pattern, while a control classification required bilateral confirmation of non-infection. Under this realistic criterion, the proposed fusion models demonstrated high stability and accuracy, particularly in the sub-lentiform nucleus. By contrast, all models showed reduced and inconsistent performance in ventricular CSF classification, further underscoring the diagnostic limitations of relying solely on ventricular analysis.

The broader implication of our findings lies in the potential of hybrid DL–radiomics frameworks to improve CNSI diagnosis in settings where LP is delayed, contraindicated, or inconclusive. To our knowledge, this is the first study to apply a deep learning–radiomics fusion approach to analyze CSF signal patterns in brain MRI for the classification of CNSIs. Prior applications of AI to fluid-based diagnostics have largely centered around cancer-related effusions or synovial fluid segmentation in rheumatology (28, 29), whereas our study opens new directions for infectious disease imaging.

DenseNet-based models consistently outperformed MobileNet-based models across most performance metrics, likely due to their deeper and densely connected architectures enabling effective feature reuse and robust representation learning. Conversely, MobileNet’s design prioritizes computational efficiency and fewer parameters, potentially limiting its capability to capture subtle radiomic patterns. Thus, DenseNet architectures may be preferable for tasks demanding detailed representation of subtle imaging features, whereas MobileNet remains beneficial under computational constraints.

Nevertheless, our study has limitations. The relatively modest sample size (n = 104) and single-center design may limit generalizability. However, all MRIs were acquired using a uniform 3 T scanner and standardized imaging protocol, enhancing internal consistency. Future research should validate these findings using multicenter datasets with larger, more diverse populations and include longitudinal evaluation across various CNSI subtypes (e.g., bacterial, viral, fungal). Additionally, the integration of clinical metadata (e.g., laboratory markers, symptoms) with imaging features may further improve classification performance. Moreover, we used a slice thickness of 5 mm, which is relatively thicker than the thin-cut images (≤3 mm) typically preferred in current brain MRI research. Although this could potentially limit the segmentation accuracy and reliability in studies utilizing 3D modeling approaches, our analyses and segmentations were strictly performed on 2D images, reducing its impact within our study context. Future studies using thinner slice imaging might offer further improvements in segmentation detail and predictive performance. Future studies could further enhance the clinical impact and interpretability of the proposed fusion models by incorporating explainable AI (XAI) methodologies to identify and visualize the most influential radiomic and deep-learning-derived features. Integrating these techniques would significantly strengthen model transparency, improve clinical confidence, and facilitate a smoother translation into clinical practice.

In conclusion, our study introduces a novel, interpretable, and clinically relevant framework for noninvasive CNSI assessment using advanced radiomics and deep learning methods. The sub-lentiform nucleus parenchyma, inclusive of PVSs, emerges as a promising anatomical region for CSF evaluation. This approach has the potential to complement traditional LP-based diagnostics and support faster, safer, and more accurate CNSI management in clinical practice.

## Data Availability

The raw data supporting the conclusions of this article will be made available by the authors, without undue reservation.
