# Supplementary material for: A novel MRI-based deep learning–radiomics framework for evaluating cerebrospinal fluid signal in central nervous system infection
Source: Front Med (Lausanne). 2025 Aug 20;12:1659653. doi: 10.3389/fmed.2025.1659653 (PMC12405214; doi:10.3389/fmed.2025.1659653)
Supplement: Supplementary file 1 [file Table_1.DOCX]

Supplementary Material

**Supplementary Table 1.** Detailed feature selection results, including Feature Name, Image Source, Feature Class, and Frequency

| **Index** | **No** | **Feature Name** | **Image Source** | **Feature Class** | **Frequency** |
| --- | --- | --- | --- | --- | --- |
| 1 | 102 | Energy | LoG-filtered MRI with fine kernel (2 mm) | First Order Statistics | 5 |
| 2 | 108 | Maximum | LoG-filtered MRI with fine kernel (2 mm) | First Order Statistics | 5 |
| 3 | 112 | Range | LoG-filtered MRI with fine kernel (2 mm) | First Order Statistics | 5 |
| 4 | 119 | Autocorrelation | LoG-filtered MRI with fine kernel (2 mm) | Gray Level Co-occurrence Matrix | 5 |
| 5 | 127 | Difference Entropy | LoG-filtered MRI with fine kernel (2 mm) | Gray Level Co-occurrence Matrix | 5 |
| 6 | 154 | High Gray Level Zone Emphasis (HGLZE) | LoG-filtered MRI with fine kernel (2 mm) | Gray Level Size Zone Matrix | 5 |
| 7 | 156 | Small Area High Gray Level Emphasis (SAHGLE) | LoG-filtered MRI with fine kernel (2 mm) | Gray Level Size Zone Matrix | 5 |
| 8 | 158 | Large Area High Gray Level Emphasis (LAHGLE) | LoG-filtered MRI with fine kernel (2 mm) | Gray Level Size Zone Matrix | 5 |
| 9 | 160 | Long Run Emphasis | LoG-filtered MRI with fine kernel (2 mm) | Gray Level Run Length Matrix | 5 |
| 10 | 173 | Long Run Low Gray Level Emphasis (LRLGLE) | LoG-filtered MRI with fine kernel (2 mm) | Gray Level Run Length Matrix | 5 |
| 11 | 174 | Long Run High Gray Level Emphasis (LRHGLE) | LoG-filtered MRI with fine kernel (2 mm) | Gray Level Run Length Matrix | 5 |
| 12 | 175 | Coarseness | LoG-filtered MRI with fine kernel (2 mm) | Neighbouring Gray Tone Difference Matrix | 5 |
| 13 | 178 | Complexity | LoG-filtered MRI with fine kernel (2 mm) | Neighbouring Gray Tone Difference Matrix | 5 |
| 14 | 189 | High Gray Level Emphasis (HGLE) | LoG-filtered MRI with fine kernel (2 mm) | Gray Level Dependence Matrix | 5 |
| 15 | 191 | Large Dependence Low Gray Level Emphasis (LDLGLE) | LoG-filtered MRI with fine kernel (2 mm) | Gray Level Dependence Matrix | 5 |
| 16 | 193 | Large Dependence Low Gray Level Emphasis (LDLGLE) | LoG-filtered MRI with fine kernel (2 mm) | Gray Level Dependence Matrix | 5 |
| 17 | 194 | Energy | LoG-filtered MRI with medium kernel (4 mm) | First Order Statistics | 5 |
| 18 | 226 | Maximal Correlation Coefficient (MCC) | LoG-filtered MRI with medium kernel (4 mm) | Gray Level Co-occurrence Matrix | 5 |
| 19 | 267 | Coarseness | LoG-filtered MRI with medium kernel (4 mm) | Neighbouring Gray Tone Difference Matrix | 5 |
| 20 | 359 | Coarseness | LoG-filtered MRI with coarse kernel (6 mm) | Neighbouring Gray Tone Difference Matrix | 5 |
| 21 | 83 | Coarseness | Original MRI | Neighbouring Gray Tone Difference Matrix | 4 |
| 22 | 105 | Minimum | LoG-filtered MRI with fine kernel (2 mm) | First Order Statistics | 4 |
| 23 | 120 | Joint Average | LoG-filtered MRI with fine kernel (2 mm) | Gray Level Co-occurrence Matrix | 4 |
| 24 | 140 | Sum Average | LoG-filtered MRI with fine kernel (2 mm) | Gray Level Co-occurrence Matrix | 4 |
| 25 | 201 | Mean | LoG-filtered MRI with medium kernel (4 mm) | First Order Statistics | 4 |
| 26 | 210 | Kurtosis | LoG-filtered MRI with medium kernel (4 mm) | First Order Statistics | 4 |
| 27 | 211 | Autocorrelation | LoG-filtered MRI with medium kernel (4 mm) | Gray Level Co-occurrence Matrix | 4 |
| 28 | 246 | High Gray Level Zone Emphasis (HGLZE) | LoG-filtered MRI with medium kernel (4 mm) | Gray Level Size Zone Matrix | 4 |
| 29 | 248 | Small Area High Gray Level Emphasis (SAHGLE) | LoG-filtered MRI with medium kernel (4 mm) | Gray Level Size Zone Matrix | 4 |
| 30 | 250 | Large Area High Gray Level Emphasis (LAHGLE) | LoG-filtered MRI with medium kernel (4 mm) | Gray Level Size Zone Matrix | 4 |
| 31 | 252 | Long Run Emphasis | LoG-filtered MRI with medium kernel (4 mm) | Gray Level Run Length Matrix | 4 |
| 32 | 265 | Long Run Low Gray Level Emphasis (LRLGLE) | LoG-filtered MRI with medium kernel (4 mm) | Gray Level Run Length Matrix | 4 |
| 33 | 266 | Long Run High Gray Level Emphasis (LRHGLE) | LoG-filtered MRI with medium kernel (4 mm) | Gray Level Run Length Matrix | 4 |
| 34 | 270 | Complexity | LoG-filtered MRI with medium kernel (4 mm) | Neighbouring Gray Tone Difference Matrix | 4 |
| 35 | 281 | High Gray Level Emphasis (HGLE) | LoG-filtered MRI with medium kernel (4 mm) | Gray Level Dependence Matrix | 4 |
| 36 | 285 | Large Dependence Low Gray Level Emphasis (LDLGLE) | LoG-filtered MRI with medium kernel (4 mm) | Gray Level Dependence Matrix | 4 |
| 37 | 71 | Run Length Non-Uniformity (RLN) | Original MRI | Gray Level Run Length Matrix | 3 |
| 38 | 103 | Total Energy | LoG-filtered MRI with fine kernel (2 mm) | First Order Statistics | 3 |
| 39 | 118 | Kurtosis | LoG-filtered MRI with fine kernel (2 mm) | First Order Statistics | 3 |
| 40 | 283 | Large Dependence Low Gray Level Emphasis (LDLGLE) | LoG-filtered MRI with medium kernel (4 mm) | Gray Level Dependence Matrix | 3 |
| 41 | 342 | Large Area High Gray Level Emphasis (LAHGLE) | LoG-filtered MRI with coarse kernel (6 mm) | Gray Level Size Zone Matrix | 3 |
| 42 | 25 | Minor Axis Length | Original MRI | Shape 2D | 2 |
| 43 | 39 | Informational Measure of Correlation (IMC) 1 | Original MRI | Gray Level Co-occurrence Matrix | 2 |
| 44 | 42 | Maximal Correlation Coefficient (MCC) | Original MRI | Gray Level Co-occurrence Matrix | 2 |
| 45 | 86 | Complexity | Original MRI | Neighbouring Gray Tone Difference Matrix | 2 |
| 46 | 107 | 90th Percentile | LoG-filtered MRI with fine kernel (2 mm) | First Order Statistics | 2 |
| 47 | 116 | Standard Deviation | LoG-filtered MRI with fine kernel (2 mm) | First Order Statistics | 2 |
| 48 | 123 | Cluster Tendency | LoG-filtered MRI with fine kernel (2 mm) | Gray Level Co-occurrence Matrix | 2 |
| 49 | 125 | Correlation | LoG-filtered MRI with fine kernel (2 mm) | Gray Level Co-occurrence Matrix | 2 |
| 50 | 142 | Sum of Squares | LoG-filtered MRI with fine kernel (2 mm) | Gray Level Co-occurrence Matrix | 2 |
| 51 | 150 | Gray Level Variance | LoG-filtered MRI with fine kernel (2 mm) | Gray Level Size Zone Matrix | 2 |
| 52 | 166 | Gray Level Variance (GLV) | LoG-filtered MRI with fine kernel (2 mm) | Gray Level Run Length Matrix | 2 |
| 53 | 185 | Gray Level Variance (GLV) | LoG-filtered MRI with fine kernel (2 mm) | Gray Level Dependence Matrix | 2 |
| 54 | 202 | Median | LoG-filtered MRI with medium kernel (4 mm) | First Order Statistics | 2 |
| 55 | 217 | Correlation | LoG-filtered MRI with medium kernel (4 mm) | Gray Level Co-occurrence Matrix | 2 |
| 56 | 224 | Informational Measure of Correlation (IMC) 2 | LoG-filtered MRI with medium kernel (4 mm) | Gray Level Co-occurrence Matrix | 2 |
| 57 | 264 | Short Run High Gray Level Emphasis (SRHGLE) | LoG-filtered MRI with medium kernel (4 mm) | Gray Level Run Length Matrix | 2 |
| 58 | 302 | Kurtosis | LoG-filtered MRI with coarse kernel (6 mm) | First Order Statistics | 2 |
| 59 | 303 | Autocorrelation | LoG-filtered MRI with coarse kernel (6 mm) | Gray Level Co-occurrence Matrix | 2 |
| 60 | 347 | Run Length Non-Uniformity (RLN) | LoG-filtered MRI with coarse kernel (6 mm) | Gray Level Run Length Matrix | 2 |
| 61 | 358 | Long Run High Gray Level Emphasis (LRHGLE) | LoG-filtered MRI with coarse kernel (6 mm) | Gray Level Run Length Matrix | 2 |
| 62 | 18 | Mesh Surface | Original MRI | Shape 2D | 1 |
| 63 | 40 | Informational Measure of Correlation (IMC) 2 | Original MRI | Gray Level Co-occurrence Matrix | 1 |
| 64 | 52 | Large Area Emphasis (LAE) | Original MRI | Gray Level Size Zone Matrix | 1 |
| 65 | 59 | Zone Variance | Original MRI | Gray Level Size Zone Matrix | 1 |
| 66 | 73 | Run Percentage | Original MRI | Gray Level Run Length Matrix | 1 |
| 67 | 115 | Root Mean Square | LoG-filtered MRI with fine kernel (2 mm) | First Order Statistics | 1 |
| 68 | 126 | Difference Average | LoG-filtered MRI with fine kernel (2 mm) | Gray Level Co-occurrence Matrix | 1 |
| 69 | 128 | Difference Variance | LoG-filtered MRI with fine kernel (2 mm) | Gray Level Co-occurrence Matrix | 1 |
| 70 | 161 | Gray Level Non-Uniformity (GLN) | LoG-filtered MRI with fine kernel (2 mm) | Gray Level Run Length Matrix | 1 |
| 71 | 195 | Total Energy | LoG-filtered MRI with medium kernel (4 mm) | First Order Statistics | 1 |
| 72 | 204 | Range | LoG-filtered MRI with medium kernel (4 mm) | First Order Statistics | 1 |
| 73 | 212 | Joint Average | LoG-filtered MRI with medium kernel (4 mm) | Gray Level Co-occurrence Matrix | 1 |
| 74 | 232 | Sum Average | LoG-filtered MRI with medium kernel (4 mm) | Gray Level Co-occurrence Matrix | 1 |
| 75 | 251 | Short Run Emphasis | LoG-filtered MRI with medium kernel (4 mm) | Gray Level Run Length Matrix | 1 |
| 76 | 253 | Gray Level Non-Uniformity (GLN) | LoG-filtered MRI with medium kernel (4 mm) | Gray Level Run Length Matrix | 1 |
| 77 | 255 | Run Length Non-Uniformity (RLN) | LoG-filtered MRI with medium kernel (4 mm) | Gray Level Run Length Matrix | 1 |
| 78 | 269 | Busyness | LoG-filtered MRI with medium kernel (4 mm) | Neighbouring Gray Tone Difference Matrix | 1 |
| 79 | 280 | Low Gray Level Emphasis (LGLE) | LoG-filtered MRI with medium kernel (4 mm) | Gray Level Dependence Matrix | 1 |
| 80 | 316 | Informational Measure of Correlation (IMC) 2 | LoG-filtered MRI with coarse kernel (6 mm) | Gray Level Co-occurrence Matrix | 1 |
| 81 | 328 | Large Area Emphasis (LAE) | LoG-filtered MRI with coarse kernel (6 mm) | Gray Level Size Zone Matrix | 1 |
| 82 | 335 | Zone Variance | LoG-filtered MRI with coarse kernel (6 mm) | Gray Level Size Zone Matrix | 1 |
| 83 | 338 | High Gray Level Zone Emphasis (HGLZE) | LoG-filtered MRI with coarse kernel (6 mm) | Gray Level Size Zone Matrix | 1 |
| 84 | 340 | Small Area High Gray Level Emphasis (SAHGLE) | LoG-filtered MRI with coarse kernel (6 mm) | Gray Level Size Zone Matrix | 1 |
| 85 | 344 | Long Run Emphasis | LoG-filtered MRI with coarse kernel (6 mm) | Gray Level Run Length Matrix | 1 |
| 86 | 351 | Run Variance (RV) | LoG-filtered MRI with coarse kernel (6 mm) | Gray Level Run Length Matrix | 1 |
| 87 | 354 | High Gray Level Run Emphasis (HGLRE) | LoG-filtered MRI with coarse kernel (6 mm) | Gray Level Run Length Matrix | 1 |
| 88 | 361 | Busyness | LoG-filtered MRI with coarse kernel (6 mm) | Neighbouring Gray Tone Difference Matrix | 1 |
| 89 | 365 | Large Dependence Emphasis (LDE) | LoG-filtered MRI with coarse kernel (6 mm) | Gray Level Dependence Matrix | 1 |
| 90 | 373 | High Gray Level Emphasis (HGLE) | LoG-filtered MRI with coarse kernel (6 mm) | Gray Level Dependence Matrix | 1 |
| 91 | 375 | Large Dependence Low Gray Level Emphasis (LDLGLE) | LoG-filtered MRI with coarse kernel (6 mm) | Gray Level Dependence Matrix | 1 |
| 92 | 377 | Large Dependence Low Gray Level Emphasis (LDLGLE) | LoG-filtered MRI with coarse kernel (6 mm) | Gray Level Dependence Matrix | 1 |
